# *Cannabis* Bioactive Compound-Based Formulations: New Perspectives for the Management of Orofacial Pain

**DOI:** 10.3390/molecules28010106

**Published:** 2022-12-23

**Authors:** Giuseppina Crescente, Giuseppe Minervini, Carmela Spagnuolo, Stefania Moccia

**Affiliations:** 1 National Research Council, Institute of Food Sciences, 83100 Avellino, Italy; 2 Multidisciplinary Department of Medical-Surgical and Dental Specialties, University of Campania, Luigi Vanvitelli, 80138 Naples, Italy

**Keywords:** *Cannabis sativa* L., cannabinoids, terpenes, orofacial pain, temporomandibular disorders, inflammation

## Abstract

The management of orofacial pain to alleviate the quality of life of affected patients is becoming increasingly challenging for scientific research and healthcare professionals. From this perspective, in addition to conventional therapies, new alternatives are being sought, increasingly looking at the use of both natural and synthetic products. *Cannabis sativa* L. represents an interesting source of bioactive compounds, including non-psychoactive cannabinoids, flavonoids, and terpenes, many of which are effective in improving pain intensity. Here, we aim to analyze the possible mechanisms of action of the bioactive natural and synthetic hemp-derived compounds responsible for the modulatory effects on pain-related pathways. The ability of these compounds to act on multiple mechanisms through a synergistic effect, reducing both the release of inflammatory mediators and regulating the response of the endocannabinoid system, makes them interesting agents for alternative formulations to be used in orofacial pain.

## 1. Introduction

The craniofacial region comprises a wide variety of tissues and annexed structures including the meninges, perioral area, temporomandibular joint (TMJ), oral mucosa, teeth, and surrounding periodontal tissues. It is richly innervated often causing acute and chronic pain [1].

Orofacial pain has been defined as a painful condition localized in the neck, face, and oral cavity and characterized by relative chronicity [2,3,4]. Trigeminal pain is especially characteristic of spinal pain in the orofacial area since it affects the trigeminal ganglion, a primary source of afferent neurons [1].

Clinically, the term chronic orofacial pain has a disputed significance, and it refers to persistent, unrelenting pain in certain areas of the face and head. The understanding of chronic orofacial pain can be simplified by separating it into two main categories: musculoskeletal pain, and neuropathic pain [2].

Approximately 33% of people suffer from myofascial pain in their face or chewing muscles, making it the second most common type of orofacial pain. It is often associated with temporomandibular joint dysfunction (TMD), affecting the chewing muscles, periauricular areas, and related structures [3,5].

Instead, it is possible to categorize the etiology of neuropathic pain into two categories: those that have a clearly defined etiology (trigeminal postherpetic neuralgia and post-traumatic trigeminal neuropathic pain), or those that are idiopathic and not determined by the underlying causes (burning mouth syndrome, persistent idiopathic facial pain, persistent idiopathic dentoalveolar pain) [6]. There are approximately 20% of acute conditions that can become chronic pain conditions if they are not treated adequately and timely. Chronic pain states are generally disabling and adversely affect the lifestyle of the affected population, causing a variety of social and psychological difficulties [5].

Due to their complexity and the limited understanding of their etiology and pathogenesis, clinical manifestations are difficult to diagnose and treat. In general, the current pain management approach combines pharmacologic medication with complementary, non-pharmacological treatments [7,8]. Still, chronic use of analgesics and anti-inflammatory drugs, as well as antidepressants used to treat chronic pain, can increase the risk of adverse drug reactions.

For this reason, research has looked towards new horizons for pain management, suggesting plant-based natural compounds as a source of bioactive molecules with interesting biological properties in this area [9,10,11,12]. The potential therapeutic benefits of natural compounds against several pain-related conditions have also been demonstrated in vivo and in vitro studies reporting their anti-inflammatory, analgesic, and antioxidant effects [10,13,14,15]. Among the different natural compounds, including polyphenols, carotenoids, and polyunsaturated fatty acids, the ability of resveratrol, lutein and docosahexaenoic acid has been demonstrated to act through the modulation of the peripheral and central nociceptive neuronal pathways, also the anti-inflammatory pathways [16].

Over the years, natural compounds have been increasingly recognized as adjuvants in the management of orofacial pain, suggesting that terpenes are the most promising agents as shown in animal models of nociception by acting through different analgesic mechanisms [10,17]. In detail, it has been shown that these compounds can act on the same systems of opioid drugs through the activation of descendent inhibitory nociceptive pathways [18]. Several studies have reported that terpenes, such as myrcene, linalool, citronellol, and citronellal, exert analgesic effects through the modulation of the opioid system and the shutdown of the inflammatory response [19,20,21].

*Cannabis sativa* L. is gaining increasing relevance in scientific research due to the content of bioactive compounds, including terpenes [21,22]. Overall, the analgesic role of cannabinoids has been demonstrated in several pain conditions, both in animal models and in clinical studies through the modulation of the endogenous cannabinoid system in mammalian nociceptive pathways [23]. A growing body of evidence supports the potential use of cannabinoid-based formulations in alleviating chronic inflammation, which underlies a variety of dysfunctions, including orofacial dysfunctions [24,25,26].

Non-psychoactive cannabinoids contribute to the pharmacological effects of hemp as reported by recent studies and show promising expectations in pain management [27,28].

Starting from these observations and considering the ability of cannabinoids to modulate pathways of orofacial nociception, it is reasonable to hypothesize that these compounds could be proposed as new analgesic agents in orofacial pain conditions [29].

Hence, we will examine the underlying molecular mechanisms by which cannabinoids can modulate pain pathways.

## 2. Mechanisms of Orofacial Pain

The neurophysiology of orofacial pain pathways consists of primary afferent neurons, pathological changes in the trigeminal ganglion, nociceptors in the brainstem, as well as higher brain functions that control orofacial pain [30].

Neurons of the trigeminal ganglion transmit damage in the orofacial region, which is perceived as orofacial pain and transmitted to the somatosensory cortex and limbic system via the spinal trigeminal nucleus and thalamic sensory nuclei [31].

**Figure 1:**
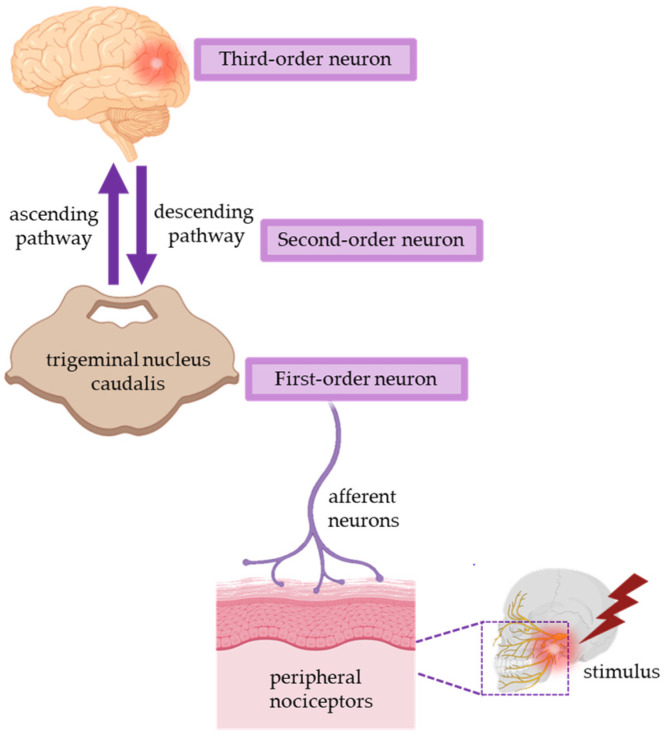
An overview of the physiology of pain in the orofacial region (Adapted from [30]).

Figure 1 provides a representation of the trigeminal pain pathway leading from the orofacial area to the brain.

In response to inflammation or damage, afferent nociceptors located in the subnucleus caudalis are responsive to these signals through a variety of physiologic and biochemical mechanisms, such as ion channels, neurokinins, and N-methyl-d-aspartate (NMDA). The subnucleus becomes more excitable, which results in allodynia, hyperalgesia, or even spontaneous pain [30].

As reported above, it is impossible to analyze all the underlying biochemical mechanisms of orofacial pain here due to the variety of types and causes of orofacial pain, so we will focus on the acute nociceptive orofacial pain and two types of chronic orofacial pain, the inflammatory TMD-related pain, and the neuropathic pain.

### 2.1. Physiological Mechanisms of Peripheral Nociceptive Pain

Acute nociceptive orofacial pain is due to harmful stimuli such as heat, cold, or mechanical and/or chemical stimuli, that directly excite nociceptive sensory neurons resulting in the transmission of pain in the central nervous system.

As reported in Figure 1, the pathophysiological mechanisms underlying the pain phenomenon are triggered by nociceptive endings that have specific receptors that convert the energy associated with mechanical, thermal, or chemical stimuli into a change in membrane potential [32,33].

One of these receptors is the transient Receptor Potential Vanilloid 1 (TRPV1), which is expressed in the sensory system and is involved in the pathophysiology of orofacial pain [34]. It is a non-selective channel for cations with excitatory activity, activated by various stimuli: thermal, chemical, and vanilloid agonists [31]. TRPV1 can be activated by various chemical mediators released after tissue injury, including K^+^ and H^+^ ions, ATP (adenosine triphosphate), leukotrienes, serotonin, and bradykinin [34]. These molecules cause the direct activation of the nociceptive terminals and the generation of action potentials through which the signal is transmitted to the central structures. Activation of nociceptors generates the Ca^2+^ current that depolarizes the distal axonal segment and triggers self-propagation of the action potential and the internal Na^+^ current [35]. Sensory afferent fiber nociceptors are activated after tissue injury and sensitized by the release of prostaglandin (PGE) synthesized by the enzyme cyclooxygenase-2 from damaged cells, bradykinin from damaged vessels, and cell mediators such as hydrogen ions and potassium [35]. The transmission in sensitized afferents recruits the release of peptides such as substance P, calcitonin gene-related peptide (CGRP), and cholecystokinin (CCK). Neuropeptides are synthesized at the level of the neuronal soma and then transferred to the terminal level by axonal transport [36]. The release of these neuropeptides leads to the degranulation of mast cells, which release histamine that can cause further activation of nociceptive terminals. In addition, nociceptive fibers can be sensitized, i.e., made more excitable, by inflammatory mediators such as PGE [32]. The pain signal generated at the peripheral branch of the nociceptor is encoded in action potentials that allow centralized transmission of the information.

### 2.2. TMJ-Related Inflammatory Pain

TMD includes several multifactorial disorders that affect the TMJ and represent the main cause of orofacial pain with several anatomical joint complications and muscle tension [37].

Often, degenerative changes in the joint can be accompanied by significant pain and dysfunction, so some individuals may have significant anterior disc displacement without pain, while others may have no evidence of degenerative changes. Sustained nociceptive input from painful temporomandibular joints appears to result in persistent sensitization of central nervous system neurons involved in ascending pain pathways.

The mechanisms underlying the nociceptive processing in the craniofacial region involved in TMJ-related pain are not fully understood. Understanding the underlying pathways and chemical mediators involved in the inflammation of the orofacial region is crucial for the management of these conditions.

In animal models of acute TMJ injury, the reduction in behavioral pain responses was associated with a decrease in joint inflammation [38].

The inflammation of the synovial membrane of the TMJ is one of the processes involved in TMD-related pain [38]. This occurs because of an imbalance of extracellular matrix synthesis and degradation in the TMJ cartilage. When the synovial membrane of TMJ is damaged, many inflammatory cytokines are produced and released in the synovial fluid and this is the key event in accentuating the destruction of cartilage and TMJ dysfunction [38]. Inflammatory biomarkers, such as pro-inflammatory cytokines, interleukins (IL), tumor necrosis factor-alpha (TNF-α), interferons, chemokines, and lymphokines, but also eicosanoids, growth factors, proteinases, and leukocyte infiltration trigger the inflammatory cascade [38]. In detail, TNF-α in the trigeminal nociceptive system has been shown to play a role in the development of TMJ inflammatory pain as reported by [39].

**Figure 2:**
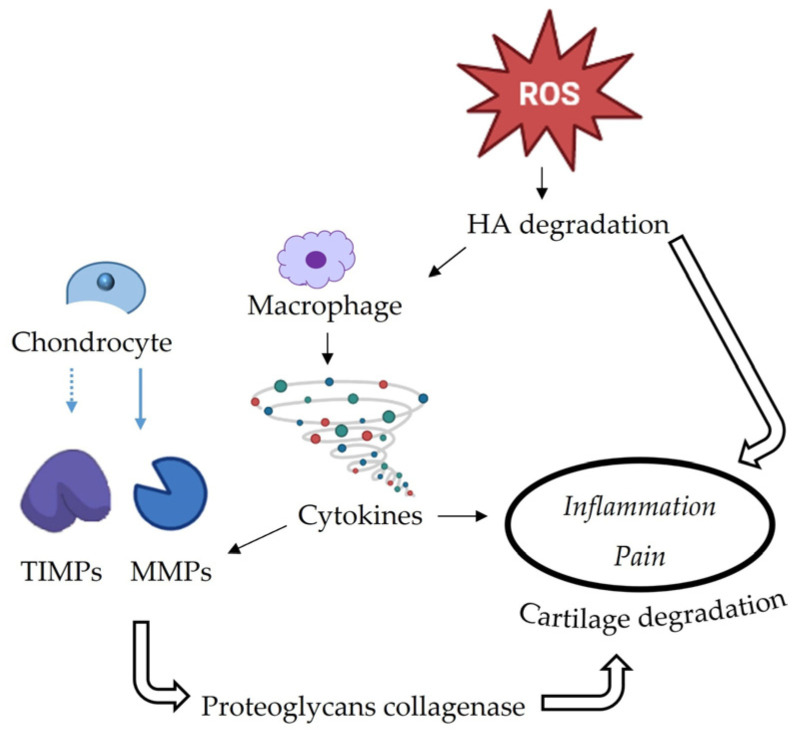
Triggered mechanisms in cartilage destruction, associated with painful TMJ (adapted from [40]).

In detail, increased production of free radicals occurs in conditions of imbalance and can lead to an impact on the degradation of hyaluronic acid (HA) which is associated with the progressive cartilage destruction occurring in TMD, as summarized in Figure 2 [40]. Chondrocytes also are stimulated to release matrix metalloproteinases (MMPs) and downregulate the tissue inhibitor of MMPs (TIMPs) involved in collagens and proteoglycans degradation.

In this context, the release of pro-inflammatory cytokines including IL-1beta, IL-8, IL-12, TNF-α, and PGE is involved in the painful symptoms, the hyperexcitability of the nociceptive pathways, and hyperalgesia [38]. In the complex setting of inflammation, pro-inflammatory cytokines are pronociceptive mediators in the trigeminal nociceptive system, leading to TMJ inflammation and trigeminal pain [41,42].

Several cellular mediators, such as hydrogen ions and potassium ions, as well as PGE released from injured cells, are produced in response to tissue damage and activate the nociceptors of sensory afferent fibers.

In particular, in vitro studies have shown that TNF-α is involved in the modulation of bone and cartilage degradation in TMD [43]. High levels of TNF-α have been detected in the synovial fluid of TMD patients and are directly associated with an increase in pain symptoms and disease stage progression [40,44]. Another study reported that cytokines promote the increase of MMPs enhancing TMJ pain and supporting the involvement of pro-inflammatory mediators in this process [40].

Researchers are increasingly focusing on discovering new ways of containing the symptoms associated with TMD-related orofacial pain since the causes of the disorder can vary widely [45,46].

Considering the just mentioned mechanisms, cytokines may therefore represent promising targets for the development of novel strategies to design new analgesic compounds [47]. However, further studies are needed to better understand the molecular mechanisms underlying inflammation in TMJ and explain the relationship between the release of pro-inflammatory cytokines and TMJ pain, and develop new therapies for TMD.

### 2.3. Pathophysiology of Neuropathic Pain

Neuropathic orofacial pain is characterized by chronic pain caused by malfunctioning somatosensory systems and nerve damage, as well as alterations in neuroplasticity [31]. Both the peripheral nervous system and the central nervous system are known to have significant roles in the progress and conservation of pain anomalies following peripheral nerve injury [32]. An injury in the central nervous system may occur after a mechanical stimulus and/or noxious compounds which stimulated the first afferent neuron, resulting in the degeneration of the myelin coating on the fibers’ distal and proximal ends (Wallerian degeneration) and the formation of neuromas [30]. Neuropathic pain can be caused by partial or mixed lesions as well as functional patterns [30]. Afferent neurons are the first ones capable of transducing the stimulus into the brain when surrounding tissues are damaged or stressed [30]. On the nociceptor level, AAE (anandamide and derivatives) releases the substance P, a neuromodulator that binds to the receptors on the side of the C fiber [32].

A neuroma or an axon in the damaged area of the trigeminal nerve increases heterotopic sodium channels, such as voltage-gated sodium channels 1.3, 1.7, and 1.8 [30]. Sodium channels propagate through the trigeminal nucleus caudalis and result in nociceptive signals which are responsible for hypersensitivity to pain [32,48].

The altered calcium channels (Ca^2+^ channels) activity in primary afferent nociceptors leads to the release of further transmitters into the trigeminal nucleus caudalis as a response to this stimulation. NMDA receptors are physiologically blocked by magnesium ions, which are known to activate trigeminal nucleus caudalis neurons [48]. As a result of the alteration of the Ca^2+^ channels, the injury inflicted on the trigeminal nucleus caudalis in the brain stem is heightened and prolonged [32].

As a consequence of nerve damage, axons and myelin sheaths are degraded, and this is followed by the infiltration of macrophages and immune cells, such as neutrophils and T cells, into the damaged area [49]. There is further functional modulation taking place in the central nervous system due to damage to the nerves [32].

## 3. *Cannabis* Bioactive Compounds

**Figure 3:**
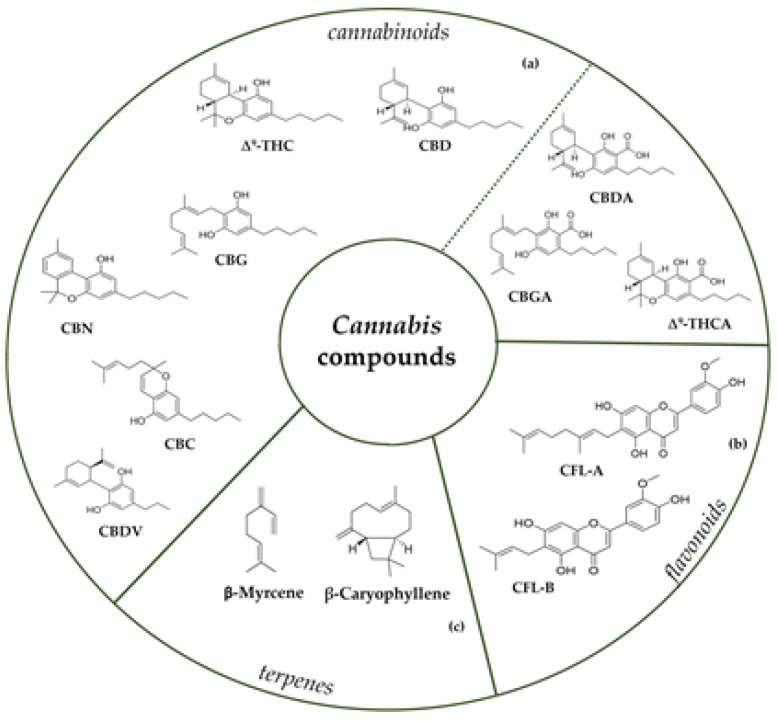
Chemical structures of main cannabinoids (**a**) both in neutral and acid form (separated by dashed line); flavonoids (**b**), as CFL-A and CFL-B; terpenes (**c**) in *Cannabis sativa* L.

*Cannabis sativa* L. represents one of the most ancient medicinal plants around the globe, which has drawn the attention of scientists worldwide [50]. It is an herbaceous plant that belongs to the Cannabaceae family and contains many secondary metabolites, including cannabinoids and non-cannabinoid-type in different parts of the plant [51,52]. Cannabinoids are C21 terpeno-phenolic specific in *Cannabis*, including Δ^9^-tetrahydrocannabinol (THC), a psychoactive compound, and cannabidiol (CBD) (Figure 3) which, unlike THC, lacks psychotropic activity, making it an attractive option for medical applications [53]; furthermore, there is also a group of minor phytocannabinoids, e.g., cannabigerol (CBG), cannabidivarin (CBDV), cannabinol (CBN), and cannabichromene (CBC) [54,55]. Indeed, the plant produces cannabinoids in the corresponding acid forms: among these, Δ^9^-tetrahydrocannabinolic acid (Δ^9^-THCA) is one of the most abundant cannabinoids present in drug-type plants, whereas the fibre-type plants contain predominantly cannabigerolic acid (CBGA) and cannabidiolic acid (CBDA) [56,57]. Acidic cannabinoids are generally decarboxylated to their neutral forms by external factors such as light, heat, and combustion [58].

*Cannabis* also contains non-cannabinoid compounds, such as non-cannabinoid phenols, flavonoids, terpenes, and alkaloids. Among flavonoids, cannflavin A (CFL-A) and cannflavin B (CFL-B) are hemp-specific methylated isoprenoid flavones (Figure 3). Hemp’s characteristic aroma is due to its terpenes. Mono- and- sesquiterpenes are detected in the aerial parts of the plant, with *β*-myrcene and *β*-caryophyllene as the most dominant compounds (Figure 3). In addition to flavonoids, hemp also contains dihydrostilbenoids, the main representative of which is canniprene. Each of the mentioned compounds has been shown to exhibit several biological effects, such as anti-inflammatory, anti-microbial, neuroprotective, and antiproliferative properties [25,50].

However, these components have not yet been studied biologically on a larger scale. Although thousands of publications have addressed the analgesic effects of psychoactive cannabinoids, somewhat fewer studies have focused on terpenes and polyphenols.

### 3.1. Cannabinoids and the Endocannabinoid System

The endocannabinoid system, together with the opioid system, represents one of the most important endogenous pain control systems and is involved in the development and maintenance of pain states as well as in the affective and cognitive aspects [59].

Cannabinoids are compounds that can interact with cannabinoid receptors (CBR), and they can be divided into (i). endogenous cannabinoids; (ii). phytocannabinoids; (iii). cannabinoids synthetics, produced for therapeutic and/or scientific research purposes [52].

The term “endogenous cannabinoids or endocannabinoids” refers to endogenous lipid messengers that interact with CBR at the central or peripheral level and regulate physiological functions. Endocannabinoids are derivatives of polyunsaturated fatty acids and include *N*-arachidonoylethanolamide (anandamide, AEA), 2-arachidonoylglycerol (2-AG), 2-arachidonyl glyceryl ether (noladine, 2-AGE), virodamine (O-arachidonoyl ethanolamine), *N*-arachidonoyl-dopamine (NADA).

Phytocannabinoids, mainly Δ^9^-THC and CBD, mimic the actions of a large family of endocannabinoid endogenous mediators, which act upon two G protein-coupled CBR: cannabinoid type-1 (CB1) and cannabinoid type-2 (CB2) which have different expression patterns due to their different physiological functions [60]. CB1 and CB2 belong to the superfamily of seven transmembrane-spanning G protein-coupled receptors and are 44% identical at the protein level [61]. The presence of CBR has been reported in both central and peripheral nervous systems and also in other tissues including bone, dental pulp, and periodontal tissues [62].

**Figure 4:**
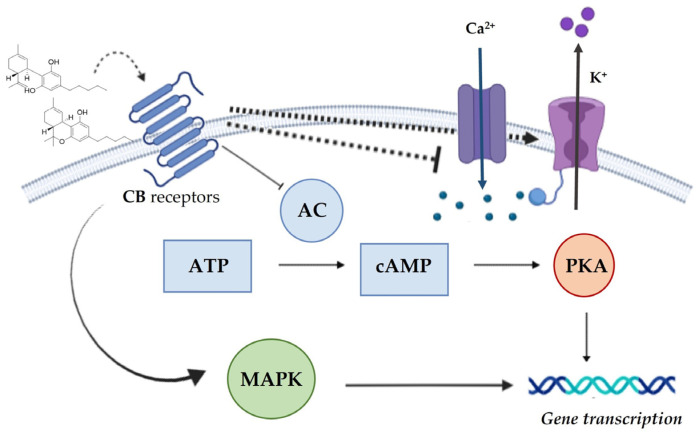
Schematic representation of cannabinoid signaling systems after ligand binding to the CBR (adapted from [63]).

The activation of the receptors involves a series of changes in cellular functions mediated by cascades of second messengers following the ligand-receptor bond. In detail, CBR signaling begins with the inhibition of adenylyl cyclase (AC), which in turn inhibits protein kinase A (PKA), by reducing ATP (Figure 4). The activation of the potassium channel (K^+^ channels) and the inhibition of the Ca^2+^ channels occur following the stimulation of the CBR as shown in Figure 4.

Activation of CB1 and CB2 leads to the inhibition of cAMP (3’,5’-cyclic adenosine monophosphate) and the activation of mitogen-activated protein kinases (MAPK) channels and thus has differential effects on cell physiology, including synaptic functions, gene transcription, cell motility, and synaptic plasticity (Figure 4) [64]. Additionally, to the stimulation of CB1 and CB2, the analgesic effects of cannabinoids may also be the result of the involvement of other neurotransmission systems including noradrenaline, serotonin, peptide systems (orexins, endorphins), as well as purinergic systems (adenosine). Cannabinoids modulate several mediators, such as glutamate and gamma amino butyric acid (GABA). Following an injury, the level of endocannabinoids increases; this occurs both locally, at the site of inflammation, and systematically on other targets of the pain pathway. Based on the type of pain, cannabinoids can act via CB1 and/or CB2 to reduce nociception [65]. Cannabinoids exert an anti-inflammatory effect through modulation of cytokine and chemokine production, modulation of adenosine signaling, expression of adhesion molecules, and migration, proliferation, and apoptosis of inflammatory cells [65]. CB1 is particularly abundant in the cortex, hippocampus, and basal ganglia, but is also expressed to a lesser extent in the hypothalamus, blood vessels, and in sympathetic nerve terminals. A large number of CB1 are located at pre-axon and axon terminals, where they regulate neurotransmitter release, and to a lesser degree at cell bodies and dendrites [66]. Detectable in trigeminal ganglions, dorsal root ganglions, and dermal nerve endings of primary sensory neurons, it regulates nociception through afferent nerve fibers. As a result, CB1 activation may trigger antinociceptive effects in peripheral nerves [67]. The activation of these receptors leads to the closing and opening of Ca^2+^ and K^+^ channels; following increased Ca^2+^ levels in the cell, 2-AG is produced intracellularly and released at the presynaptic terminal, where it binds to CB1. Through the inhibition of Ca^2+^ channels, presynaptic Ca^2+^ influx is reduced, suppressing neurotransmitter release [66]. CB2, on the other hand, is usually expressed primarily by cells of the immune system, including glial cells of the central nervous system. However, the activity of CB2 on neurons has been shown to have functional effects in more recent studies [60]. Studies in the past decade have suggested that there are additional CBR apart from CB1 and CB2. A putative cannabinoid 3 receptor is G protein-coupled receptor 55 (GPR55). The amino acid sequence of GPR55 shows only a low degree of identity to CB1 (13.5%) and CB2 (14.4%), but it shares several cannabinoid ligands. There is a large distribution of GPR55 in the body, and its role has been suggested in numerous physiological processes as well as pathophysiological processes, including gut pathophysiology, inflammation, and neuropathic pain, as well as modulating the innate and adaptive immune systems [68]. TRPV1, a second CBR, is also known as the capsaicin receptor and has been called an ionotropic CBR by many scientists. When activated, TRPV1 induces the release of the substance P and CGRP, which play an important role in pain detection and tissue inflammation [69].

### 3.2. Cannabis-Based Formulations as Orofacial Pain Relievers

Studies investigating the effects of natural and synthetic *Cannabis*-based formulations on acute nociceptive pain, TMJ-related inflammatory pain, and chronic neuropathic pain were included in the review. We considered in vivo studies (human and animal models), systematic reviews, and meta-analyses that involved the use of *Cannabis* and its derivatives, both natural and synthetic, as interventions vs. placebo through different routes of administration (topical and oral). Research on *Cannabis*-based products’ effects on the orofacial region is lacking; other evidence, however, supports their use in treating chronic nociceptive and neuropathic pain in other areas [24]. Phytocannabinoids share a similar chemical structure, however, their pharmacological effects may differ [70]. Despite being able to provide limited information regarding cannabinoids’ pharmacokinetics and pharmacodynamics, different pharmacokinetic effects were observed based on the formulation and route of administration [71]. The oral bioavailability of cannabinoids is poor, which has led to the development of effective administration techniques, such as transdermal administration, intranasal administration, and transmucosal adsorption [70]. Both THC and CBD have high lipophilicity, which means that they should be more effective at absorbing into the skin [71,72]. Furthermore, they would be good candidates for advanced nanosized drug delivery systems [70]. In a study by [72] CBD cream’s effectiveness has been evaluated in patients with TMD [72]. In detail, the test hemp oil containing CBD was incorporated into a formulation based on cholesterol ointment: patients were required to apply it twice daily to the skin surface for 14 days. Patients suffering from myofascial pain benefited from formulation application over masseter muscles improving masticatory muscle condition concerning the control group [72]. In myofascial pain therapy, transdermal administration via patches may be very useful. Consequently, oral administration is avoided as well as intramuscular injections, resulting in better blood absorption because first-pass metabolism is not encountered [72]. Evidence that CBD reduces cytokine release has been confirmed by in vivo studies [73]. In detail, CBD inhibiting GPR55 leads to the inactivation of its pronociceptive signaling [72].

In cutaneous sensory nerve endings, capsaicin receptor TRPV1 may also be desensitized by cannabinoids, enhancing thus the analgesic activity [74]. Studies have shown that CBR activation leads to the phosphorylation of TRPV1 as a result of intracellular signaling cascade activation [75]. When CBR are activated, keratinocytes release endogenous opioids which reduce pain in the epidermis [76].

In another study conducted by [77], eucalyptol, a monoterpene present in *Cannabis*, exerts antinociceptive properties against orofacial pain in rodent models [77]. Using eucalyptol oral pre-treatment for 60 min, they observed that it reduces spontaneous pain behaviors induced by formalin, capsaicin, and glutamate injected into the lip, formalin, and mustard oil into the temporomandibular joint, and hypertonic saline into the cornea. The mechanism of eucalyptol-induced antinociception demonstrated an interaction between eucalyptol and TRPV1 receptors, inhibiting it [77].

The studies mentioned above with their relative characteristics have been summarized in Table 1.

### 3.3. Synthetic Cannabinoids Agonist Receptor-Based Formulations as Orofacial Pain Relievers

Initially designed to explore the endogenous cannabinoid system, synthetic cannabinoids were approved for analgesia in humans [17]. Despite not sharing the same chemical structure as cannabinoids, they agonize CB1 and CB2 and typically have a greater affinity for the CB1 than endocannabinoids [78].

WIN 55,212-2, a cannabinoid agonist, was evaluated to investigate the antinociceptive effect in the orofacial and temporomandibular formalin tests; WIN was administered to rats (0.5, 1 mg/kg) 30 min before formalin was injected: at a dose of 1 mg/kg induced an antinociceptive effect in both peri-orofacial models equivalent to a dose of 10 mg/kg morphine (*p* < 0.001 vs. control) [29]. This analgesic effect can be attributed to its specific action on the CB1 in the orofacial and TMJ regions [29]. According to the results of [29], it has been shown that WIN, when administered intracisternally, induces antinociception in the TMJ formalin test via CB1 activation [29]. Furthermore, in agreement with [79], CB1 antagonists selectively block the antinociceptive effect induced by WIN in a rat model of trigeminal neuropathic pain suggesting that cannabinoids’ antinociceptive effects on orofacial pain are driven by CB1 rather than CB2 [79]. Instead, the oral administration of two synthetic CBR agonists (AZD1940 and GW842166) did not produce significant analgesic effects in two studies performed after administering a single cannabinoid dose (AZD1940 800 mg, GW842166 800 mg) between 1 to 1.5 h before the surgical third molar removal [80,81].

The studies mentioned above with their relative characteristics have been summarized in Table 2.

## 4. Discussion

Orofacial pain disorders are widespread and debilitating conditions that affect the head, face, neck, teeth, and their supporting structures, TMJ, and muscles of the head [33]. For this reason, pain is a common symptom, and the complexity of these structures makes it difficult to recognize that they may be migraine, TMD, and trigeminal neuropathies and difficult to treat.

Improving knowledge of the mechanisms of several pain conditions may allow diagnosis through a multidisciplinary approach. In general, the most debilitating pain conditions emanate from the structures innervated by the trigeminal system (head, face, masticatory muscles, temporomandibular joint, and associated structures) and involve both peripheral and central amplification of nociceptive signals that may maintain pain [36].

This work is inspired by the growing evidence that supports the use of hemp in pain management. In particular, there are many studies supporting the ability of *Cannabis*-based products to ameliorate symptoms associated with chronic inflammation and neuropathic pain, both conditions that can be related to a variety of pathologies.

A recent systematic review on *Cannabis* and orofacial pain were conducted by [24], who critically analyzed the effects of *Cannabis* and its naturally and synthetically derived products in relieving orofacial pain and inflammation [24].

In detail, promising expectations emerged in the topical application of cannabidiol-based formulations through the implementation of muscle function resulting in the improvement of symptoms in patients with myofascial pain [24]. Another recent systematic review with meta-analysis has carefully investigated the use of *Cannabis*-based medications in chronic neuropathic pain patients, suggesting a significant impact on the perception and intensity of pain following THC and THC/CBD interventions compared to placebo [82]. However, the potential benefit and actual application of formulations based on cannabinoids, oral supplementation, and oromucosal spray depend on the high variability and exclusion criteria of the study participants [82]. Here we focus on studying the mechanisms related to the modulation of orofacial pain by several bioactive compounds present in hemp, including terpenes. As previously discussed, *Cannabis* has up to 200 different terpenes, which are responsible for the varied aromas, flavors, and other characteristics of different strains [21]. Known as lipophilic molecules, they act at a wide range of sites, including neurotransmitter receptors, muscle, and neuronal ion channels, G-protein receptors, enzymes, cell membranes, and second messenger systems [83]. Usually, their pharmacological properties have been studied mainly relating to their potential analgesic action, supporting their role as candidates for the treatment of several pathological conditions, including pain-related conditions [82]. In this regard, the anti-inflammatory effects of many compounds such as citral, *α*-terpineol, and *α*-pinene are due to their ability to modulate the nuclear factor kappaB (NF-kB) pathway, an important signaling pathway involved in the development of inflammatory diseases, and to decrease the synthesis and release of nitric oxide (NO), as shown in vivo and in vitro studies [84]. In particular, *α*-pinene has been found to have anti-inflammatory properties, as well as anti-inflammatory properties via PGE-1. Menthol features antipruritic properties and is an analgesic used topically to treat joint and muscle pain [85]. Analgesia involves activating sensory neurons at the transient receptor potential melastatin 8 (TRPM8) receptor and selectively activating kappa-opioid receptors. Constriction of sodium and Ca^2+^ channels is inhibited [21]. There is also evidence that terpenes can increase the blood-brain barrier permeability and have been used as a permeation agent in a patent for a transdermal cannabinoid patch [86]. The therapeutic potential of other classes of natural compounds including polyphenols has been studied in reducing pain and inflammation in spinal cord injury, evidencing also other mechanisms of action [87]. Approximately 20 flavonoids have been identified in *Cannabis* [21]. These include apigenin, luteolin, quercetin, kaempferol, cannflavin A, cannflavin B (specific to *Cannabis*), as well as vitexin, isovitexin, and kaempferol [86]. The anti-inflammatory, neuroprotective, and anti-cancer effects of many of these compounds are similar to those of terpenes. As well as having anxiolytic properties, apigenin inhibits TNF-α 931, which is implicated in many inflammatory conditions. It has been found that cannflavin A and B have potent anti-inflammatory properties; in detail, cannflavin A inhibits PGE-2 30 times more effective than aspirin [21]. On the other, phenolic compounds found in *Cannabis* have had much less research conducted. To make another example, green tea polyphenol extract has been shown as a complementary treatment for pain control in fifty adults with knee osteoarthritis (OA) after four weeks of supplementation [88]. Red berry polyphenols have also shown significant benefits in managing the symptoms and progression of arthritis, including joint pain, in a randomized, double-blind cross-over trial [89]. Specifically, strawberry drink administration (50 g/day) over 12 weeks, significantly reduced serum biomarkers of inflammation and cartilage degradation such as IL-6, IL-1β, and MMPs-3 in 17 adults with OA of the knee [89]. On the other hand, pure phenolics, such as curcumin, have also been shown to exert a modulatory role in pain and inflammation by inhibiting proinflammatory mediators, metabotropic glutamate receptor-2 (mGlu2) and the monoamine system, and regulating the opioid system [90]. A recent interesting study supported the role of resveratrol (40 mg/kg or 80 mg/kg) in alleviating inflammatory TMJ pain by restoring the gut microbiome in the TMJ inflammation mouse model [91]. Indeed, the involvement of gut microbiome alterations in the development of TMJ inflammation has been described, suggesting a new therapeutic approach for the treatment of TMJ pain [91]. Recent studies are aimed in this direction, proposing a role of the endocannabinoid system in the microbiota-gut-brain axis, which could play a key role in the control of cognitive functions and possible pathological alterations [92]. In light of these new assumptions, it is reasonable to hypothesize that the endocannabinoid system may also contribute to the regulation of transmission in orofacial pain and that it may represent a potential target for future studies.

**Figure 5:**
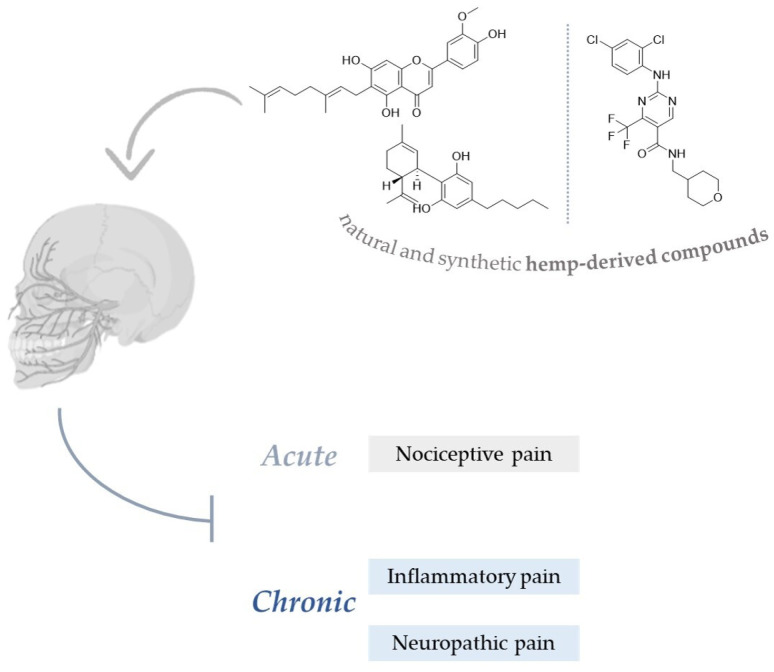
Pleiotropic mechanisms triggered by bioactive compounds of *Cannabis sativa* L. involved in the onset of inflammatory pain in TMJ.

This is further reinforced by the assumption that CBR is present in several tissues, it is reasonable to assume that the different bioactive compounds of hemp can interact with the signaling pathways of orofacial pain in the reduction of periodontal, myofascial, and TMJ pain. The ability of cannabinoids to act as pain relievers through multiple mechanisms suggests that the bioactive compounds of hemp, including phytocannabinoids, terpenes, and flavonoids could act synergistically by modulating the inflammatory pathway and targeting multiple mechanisms involved in the onset of TMJ-orofacial pain, as summarized in Figure 5.

## 5. Conclusions

This review aims to elucidate the mechanisms by which bioactive natural and synthetic hemp compounds can block the excitability of trigeminal nociceptive neurons, resulting in the modulation of the intensity of TMJ-related orofacial pain. Among the different mechanisms analyzed, the various bioactive compounds in hemp, including phytocannabinoids, flavonoids, and terpenes, were found to potentially act through a synergistic effect that makes them ideal candidates for pain relief in nociceptive and neuropathic pain. It should also not be underestimated that the pleiotropic action of these compounds involves the modulation of signaling pathways through various mechanisms, which include the reduction of the inflammatory response, which is one. Future studies will aim to understand the mechanisms of action and develop new formulations focusing major attention also on the other bioactive not fully considered hemp compounds that can be used in the management of orofacial pain.

## Figures and Tables

**Table 1:** An overview of the included studies’ characteristics using natural compounds: in panel (**a**) the studies conducted on humans and (**b**) those on animal models are included.

**(a)**				
**Formulation**	**Dose and Route/Time** **of Administration**	**Study Participants**	**Results**	**Reference**
CBD formulation	Topical/twice a day for 14 days	60 patients (Female/male)	Reduction in masseter activity; decrease of pain intensity according to VAS scale; improvement of muscle function in patients with myofascial pain	[72]
**(b)**				
**Formulation**	**Dose and route/time** **of administration**	**Animal models**	**Results**	**Reference**
Eucalyptol	100, 200, and 400 mg/kg; Single dose 1 h before the induction	Male Swiss, C57BL/6 J or BALB/c mice; 20–25 g;	Reduction of face rubbing time on capsaicin and glutamate tests	[77]

**Table 2:** An overview of the included studies’ characteristics using synthetic cannabinoid agonist receptors: in panel (**a**) the studies conducted on humans and (**b**) those on animal models are included.

**(a)**				
**Formulation**	**Dose and Route/Time of Administration**	**Study Participants**	**Results**	**Reference**
Agonist of CBR-2 (GW842166)	100 mg; Single dose 1 h before the surgical third molar removal	34 patients (Female/male)	Similar effect to the placebo	[81]
Agonist of CBR-2 (GW842166)	800 mg; Single dose 1 h before the surgical third molar removal	27 patients (Female/male)	Improvement in analgesia over placebo with both VAS and VRS pain ratings	[81]
Cannabinoid agonist (AZD1940)	800 μg; Single dose 1.5 h before the surgical third molar removal	61 patients (Female/male)	No statistically significant difference in pain compared with the placebo	[80]
**(b)**				
**Formulation**	**Dose and route/time of administration**	**Animal models**	**Results**	**Reference**
Cannabinoid agonist (WIN 55,212-2)	Intraperitoneal; 0.5, 1 mg/kg; Single dose 30 min before the nociceptive stimulus	Adult male Wistar rats; weight: 200–250 g	Antinociceptive effect	[29]

## Data Availability

Not applicable.

## Abbreviations

TMJ	temporomandibular joint
TMD	temporomandibular disorders
TRPV1	transient receptor potential vanilloid 1
ATP	adenosine triphosphate
PGE	prostaglandin
CGRP	gene-related peptide calcitonin
CCK	cholecystokinin
IL	interleukin
TNF-α	tumor necrosis factor-alpha
HA	hyaluronic acid
MMPs	matrix metalloproteinases
TIMPs	tissue inhibitor of metalloproteinases
THC	Δ^9^-tetrahydrocannabinol
Δ^9^-THCA	Δ^9^-tetrahydrocannabinolic acid
CBD	cannabidiol
CBDA	cannabidiolic acid
CBG	cannabigerol
CBGA	cannabigerolic acid
CBDV	cannabidivarin
CBN	cannabinol
CBC	cannabichromene
CFL-A	cannflavin A
CFL-B	cannflavin B
CBR	cannabinoid receptors
AEA	*N*-arachidonoylethanolamide
2-AG	2-arachidonoylglycerol
2-AGE	2-arachidonyl glyceryl ether
NADA	*N*-arachidonoyl-dopamine
CB1	cannabinoid receptor 1
CB2	cannabinoid receptor 2
AC	adenylyl cyclase
PKA	protein kinase A
K^+^ channel	potassium channel
Ca^2+^ channel	calcium channel
cAMP	3’,5’-cyclic adenosine monophosphate
MAPK	mitogen-activated protein kinases
GABA	γ-aminobutyric acid
GPR55	G protein-coupled receptor 55
PGE	prostaglandin
TRPM8	transient receptor potential melastatin 8
NF-KB	nuclear factor kappaB
NO	nitric oxide
OA	osteoarthritis
mGlu2	metabotropic glutamate receptor 2
NMDA	N-methyl-d-aspartate
AAE	anandamide and derivatives
